# Trends of Racial/Ethnic Differences in Emergency Department Care Outcomes Among Adults in the United States From 2005 to 2016

**DOI:** 10.3389/fmed.2020.00300

**Published:** 2020-06-25

**Authors:** Xingyu Zhang, Maria Carabello, Tyler Hill, Sue Anne Bell, Rob Stephenson, Prashant Mahajan

**Affiliations:** ^1^Department of Systems, Populations, and Leadership, University of Michigan School of Nursing, Ann Arbor, MI, United States; ^2^Department of Health Management and Policy, University of Michigan School of Public Health, Ann Arbor, MI, United States; ^3^Department of Psychology, College of Literature, Science, and the Arts, University of Michigan, Ann Arbor, MI, United States; ^4^Department of Emergency Medicine, University of Michigan School of Medicine, Ann Arbor, MI, United States

**Keywords:** health disparities, African-American, emergency care, health outcomes, resource utilization, trend

## Abstract

**Importance:** While the literature documenting health disparities has advanced in recent decades, less is known about the pattern of racial/ethnic disparities in emergency care in the United States.

**Objective:** To describe the trends and differences of health outcomes and resource utilization among racial/ethnic groups in US emergency care for adult patients over a 12-year period.

**Design, Setting, and Participants:** This cross-sectional study of emergency department (ED) data from the nationally representative National Hospital Ambulatory Medical Survey (NHAMCS) examined multiple dimensions of ED care and treatment from 2005 to 2016 among adults in the US.

**Main Outcomes and Measures:** The main outcomes include ED care outcomes (hospital admission, ICU admission, and death in the ED/hospital), resource utilization outcomes (medical imaging use, blood test, and procedure use), and patients' waiting time in the ED. The main exposure variable is race/ethnicity including white patients (non-Hispanic), black patients (non-Hispanic), Hispanic patients, Asian patients, and Other.

**Results:** During the 12-year study period, NHAMCS collected data on 247,989 adult (> 18 years old) ED encounters, providing a weighted sample of 1,065,936,835 ED visits for analysis. Asian patients were 1.21 times more likely than white patients to be admitted to the hospital following an ED visit (aOR 1.21, 95% CI 1.12–1.31). Hispanic patients presented no significant difference in hospital admission following an ED visit (aOR 1.01, 95% CI 0.97–1.06) with white patients. Black patients were 7% less likely to receive an urgent ESI score than white patients less likely to receive immediate or emergent scores, as opposed to semi- or non-urgent scores. Black patients were also 10% less likely than white patients to be admitted to the hospital and were 1.26 times more likely than white patients to die in the ED or hospital.

**Conclusions and Relevance:** Race is associated with significant differences in ED treatment and admission rates, which may represent disparities in emergency care. Hispanic and Asian Americans were equal or more likely to be admitted to the hospital compared to white patients. Black patients received lower triage scores and higher mortality rates. Further research is needed to understand the underlying causes and long-term health consequences of these disparities.

## Introduction

Nearly two decades ago, the Institute of Medicine (IOM) released a historic report documenting significant racial and ethnic disparities in the US healthcare delivery system, along with policy recommendations to address their findings (1). The IOM report noted that minority groups, including black and Hispanic populations, face critical differences in healthcare access owing to higher rates of uninsurance, reduced choice in where to receive care, and a variety of structural, cultural, and linguistic barriers as compared to white people (2). Further, black and Hispanic individuals are less likely than whites to have a primary care provider for routine and preventive health needs and are more likely to seek care in a hospital emergency department (ED) (2–4).

Consistent with these findings, the ED is more likely to serve as the entry point into the healthcare system for racial and ethnic minorities than it is for white populations in the US (5, 6). Upon arrival to the ED, minority populations have also been found to receive disparate treatment for a number of common symptoms, including chest pain and acute coronary events; (7–11) trauma; (12–14) stroke symptoms and brain injuries; (15, 16) and pain management for bone fractures, migraines, and back pain as compared to white patients (17–19). Such disparities are alarming in light of the strong association between emergency care quality and mortality risk and the heightened threat of racial biases affecting providers' decision-making in the fast-paced, information-poor ED context (20).

In seeking to explore and document racial and ethnic disparities in ED care and outcomes, we and prior researchers recognize the nature of race and ethnicity as socially-constructed categories that have real consequences for the experiences and life chances of people of color within US society writ large (21), and also within the US healthcare system. In such studies, race and ethnicity are used as proxies for the true underlying determinant of the observed disparities, which is racism (22). As an entrenched and hierarchical system of stratification, racism structures how minority groups currently and historically have faced differential access to resources, opportunities, and risks, making it a fundamental cause of health and health disparities (21, 23). Documenting and seeking to eliminate racial and ethnic disparities in emergency care represents a crucial step in ongoing efforts to create a more equitable healthcare system for all patients in the United States (24), and will require further research and intervention into the broader systems of stratification that seed sources of inequity within our health and social systems. To help inform these efforts, we examined patterns in ED care outcomes and utilization rates for Asian, black, Hispanic, and non-Hispanic white adults and investigated factors that may contribute to observed care disparities, using a nationally representative dataset for US ED visits.

## Methods

This is a cross-sectional study of ED data obtained from a multiyear, nationally representative survey carried out in the US. This study used preexisting, de-identified data and thus was categorized as exempt by the University of Michigan's Institutional Review Board.

### Data Source and Study Population

The study population was derived from the National Hospital Ambulatory Medical Survey (NHAMCS) Emergency Department Subfile (NHAMCS-ED) between 2005 and 2016 (25). NHAMCS-ED is a multistage, stratified probability sample of ED visits in the US, administered by the National Center for Health Statistics (a branch of the Centers for Disease Control and Prevention) (26). The NHAMCS-ED sample is collected from ~300 hospital-based EDs per year, randomly selected from ~1,900 geographic areas in all 50 states. The survey uses a standardized data collection form to capture detailed information from ~100 patients per hospital-based ED. A total of 358,163 patient (Weighted *N* = 1,560,846,342) visits from 3,764 hospital-based EDs were included in the survey datasets from 2005 to 2016. To restrict our sample to adult patients with one documented race/ethnicity, we excluded all pediatric visits (age <18 years, *n* = 81,452, Weighted *N* = 354,288,756) and patients with unknown or multiple races (*n* = 28,722; Weighted *N* = 140,620,751), resulting in a total of 247,989 (Weighted *N* = 1,065,936,835) patients visits for analysis.

### Study Outcomes

The primary study outcome variables include the triage level [emergency severity index [ESI], a five-level ED triage algorithm providing clinically relevant stratification of patients into five groups from 1 [most urgent] to 5 [least urgent] on the basis of visit acuity level and resource needs], hospital admission, intensive care unit (ICU) admission, death in the ED/hospital, medical resources utilization (blood test, imaging, and other procedures; see Supplement Table 1 for a full), waiting time (time between arrival and seeing a physician), and length of visit (time from arrival to discharge) for the ED encounter. Death outcomes include deaths in the ED and deaths in the hospital. The primary exposure variables for the analysis was a patient's racial/ethnic categorization. Race was predefined by NHAMCS as Asian, black, white, or other (including those who identify as American Indian/Alaska native and Native Hawaiian/Other Pacific Islander), and ethnicity was categorized as either Hispanic or non-Hispanic. From these categorizations we arrived at the following mutually exclusive groups for analysis: non-Hispanic black (hereafter referred to as black), Hispanic, Asian, other, and non-Hispanic white (hereafter referred to as white), as the baseline for comparison. For simplicity, we refer to these as racial groups throughout the remainder of the manuscript; all non-white categories are considered racial minorities for the purposes of our analysis. Given the small sample size and heterogeneity of those racially-classifed as “other,” we report data for this group in the tables but do not focus on them in our discussion.

For adjusted analyses, we included patient demographic variables (sex and age group) and variables indicative of socioeconomic status, including residence type (private home, nursing home, homeless, or other), insurance type (private insurance, Medicare, Medicaid/CHIP, uninsured, or other), arrival mode, arrival day of the week, and time category. We also included clinical variables such as triage vital signs (body temperature, heart rate, diastolic blood pressure, and pain scale). Additional patient-level covariates included whether or not the patient had visited the ED within the past 72 h. We also included information on the US census region of the ED and the primary reason for the ED visit based on system-based symptom clusters (e.g., “symptoms referable to the respiratory system”). We used these symptom clusters as they were present in the dataset for the entire patient population.

### Statistical Analysis

Population characteristics were described and compared among different racial/ethnic groups. The proportion of each outcome variable among different racial/ethnic groups and covariate groups were compared using chi-square tests. Multivariable logistic regression models were used to estimate the association between each binary outcome (hospital admission, ICU admission, death, blood test, medical imaging utilization, and procedure use) and racial/ethnic groups. Multinomial logistic regression models were used to estimate the association between the ESI scores (categorical) and racial/ethnic groups. Models were sequentially adjusted for demographic, socioeconomic, and visit/clinical variables; specifically, we adjusted for ESI scores to test for changes in the associations between racial/ethnic group and binary outcomes. Multivariable linear regression was used to test the association between the waiting time and length of visit (continuous variables) and racial/ethnic groups after adjusting for other confounding variables. Because these two variables are not normally distributed, a log transformation was performed prior the regression model. Poisson regression was used to model trends in the outcome rates among racial/ethnic groups, adjusting for age, gender, and insurance type, with time modeled linearly as years since 2005. An interaction between the racial/ethnic group and time was included in each model to test whether the trend in each outcome differed by racial/ethnic group.

The NHAMCS-ED dataset used in this analysis relies on imputation for missing data. Specifically, the survey uses a hot deck-based single, sequential regression methodology to impute 3-digit ICD-9-CM codes for items such as age, sex, primary diagnosis, ED volume, and geographic region. The other variables were imputed with the median of the corresponding variables prior to generating the logistic regression models and multivariable linear regression models. SAS (version 9.4) was used for analyses, and alpha = 0.05 was set as the statistical significance threshold.

## Results

During the 12-year study period between 2005 and 2016, NHAMCS collected data on 247,989 adult (> 18 years old) ED encounters with a discrete race categorization, providing a weighted sample of 1,065,936,835 for analysis (Table 1 and Supplement Table 2). The analysis was stratified by racial/ethnic groups in the following proportions: white patients, 64.3%; black patients, 22.1%; Hispanic patients, 11.0%; Asian patients, 1.7%; and other, 0.01%. Rates of uninsurance were highest for Hispanic patients (24.2%) and black patients (22.4%) and lowest for white (15.2%) and Asian patients (13.7%). Compared to Asian and white patients, a greater proportion of black patients, Hispanic patients, and other racial/ethnic minority ED patients belonged to the 18–39 age group. In terms of symptoms, black patients presented with the highest proportion of respiratory issues (11.6% of visits), and Hispanic patients presented with the highest proportion of digestive issues (18.4% of visits).

**Table 1:** Baseline characteristics of patients presenting to the ED, stratified by race/ethnicity, NHAMCS 2005–2016 (weighted sample).

	**All**	**White patients**	**Black patients**	**Hispanic patients**	**Asian patients**	**Other**
	1,065,936,835	684,948,994 (64.3)	235,356,879 (22.1)	116,893,764 (11.0)	18,123,016 (1.7)	10,614,182 (1.0)
**Male**	458,688,091 (43.0)	300,429,341 (43.9)	95,120,971 (40.4)	50,676,958 (43.4)	7,945,064 (43.8)	4,515,757 (42.5)
**Age**						
18–39	454,826,892 (42.7)	267,506,162 (39.1)	114,288,373 (48.6)	60,799,082 (52.0)	7,398,866 (40.8)	4,834,409 (45.5)
40–49	180,241,636 (16.9)	111,092,537 (16.2)	44,013,302 (18.7)	20,476,127 (17.5)	2,513,301 (13.9)	2,146,369 (20.2)
50–59	157,552,708 (14.8)	100,746,731 (14.7)	37,331,253 (15.9)	15,295,944 (13.1)	2,594,595 (14.3)	1,584,185 (14.9)
60–74	149,354,632 (14.0)	105,915,810 (15.5)	26,418,286 (11.2)	12,708,516 (10.9)	2,933,089 (16.2)	1,378,930 (13.0)
≥ 75	123,960,967 (11.6)	99,687,753 (14.6)	13,305,663 (5.7)	7,614,095 (6.5)	2,683,166 (14.8)	670,289 (6.3)
**Residence type**						
Private residence	975,224,202 (95.1)	622,404,310 (94.6)	218,576,189 (95.9)	107,770,949 (96.2)	16,659,256 (96.3)	9,813,496 (94.8)
Nursing home	25,715,854 (2.5)	20,136,772 (3.1)	3,770,466 (1.7)	1,342,768 (1.2)	368,971 (2.1)	96,877 (0.9)
Homeless	7,801,609 (0.8)	4,396,947 (0.7)	1,940,447 (0.9)	1,147,133 (1.0)	84,241 (0.5)	232,841 (2.2)
Other	16,683,842 (1.6)	10,798,624 (1.6)	3,717,765 (1.6)	1,771,526 (1.6)	186,037 (1.1)	209,890 (2.0)
**Insurance type**						
Private insurance	321,335,956 (32.0)	226,685,656 (34.8)	57,394,147 (26.2)	27,602,519 (25.7)	6,798,262 (40.0)	2,855,372 (28.0)
Medicare	243,754,059 (24.3)	184,553,685 (28.4)	39,850,264 (18.2)	14,215,845 (13.2)	3,418,729 (20.1)	1,715,536 (16.8)
Medicaid or CHIP	218,162,456 (21.7)	113,269,715 (17.4)	64,314,663 (29.4)	33,678,741 (31.3)	3,714,171 (21.8)	3,185,167 (31.3)
Uninsured	178,164,973 (17.7)	98,987,765 (15.2)	49,073,069 (22.4)	26,032,882 (24.2)	2,337,085 (13.7)	1,734,173 (17.0)
Other	43,012,416 (4.3)	27,293,947 (4.2)	8,288,832 (3.8)	5,997,610 (5.6)	734,364 (4.3)	697,663 (6.8)
**Year**						
2005	82,947,472 (7.8)	54,395,817 (7.9)	16,112,439 (6.8)	10,066,446 (8.6)	1,606,437 (8.9)	766,333 (7.2)
2006	90,593,915 (8.5)	56,734,743 (8.3)	21,008,698 (8.9)	10,121,898 (8.7)	1,748,174 (9.6)	980,402 (9.2)
2007	78,675,398 (7.4)	51,657,544 (7.5)	17,658,282 (7.5)	7,564,031 (6.5)	1,156,816 (6.4)	638,725 (6.0)
2008	81,178,690 (7.6)	53,715,245 (7.8)	18,223,694 (7.7)	7,254,941 (6.2)	1,225,025 (6.8)	759,785 (7.2)
2009	93,571,540 (8.8)	60,455,172 (8.8)	21,576,760 (9.2)	8,547,722 (7.3)	1,929,975 (10.6)	1,061,911 (10.0)
2010	92,276,613 (8.7)	60,909,002 (8.9)	19,058,853 (8.1)	9,953,062 (8.5)	1,512,008 (8.3)	843,688 (7.9)
2011	93,739,810 (8.8)	59,512,636 (8.7)	21,647,273 (9.2)	9,922,551 (8.5)	1,640,597 (9.1)	1,016,753 (9.6)
2012	87,134,529 (8.2)	54,874,117 (8.0)	18,995,723 (8.1)	10,664,317 (9.1)	1,605,220 (8.9)	995,152 (9.4)
2013	87,119,811 (8.2)	56,919,172 (8.3)	18,411,529 (7.8)	9,407,125 (8.0)	1,306,551 (7.2)	1,075,434 (10.1)
2014	90,554,699 (8.5)	54,674,408 (8.0)	22,597,298 (9.6)	10,798,049 (9.2)	1,620,620 (8.9)	864,324 (8.1)
2015	89,005,064 (8.3)	56,828,676 (8.3)	19,634,219 (8.3)	10,709,406 (9.2)	1,243,528 (6.9)	589,235 (5.6)
2016	99,139,294 (9.3)	64,272,462 (9.4)	20,432,111 (8.7)	11,884,216 (10.2)	1,528,065 (8.4)	1,022,440 (9.6)
**Day of Week**						
Sunday	146,504,562 (13.7)	96,204,439 (14.0)	30,683,285 (13.0)	15,512,945 (13.3)	2,536,509 (14.0)	1,567,383 (14.8)
Monday	168,098,140 (15.8)	106,063,337 (15.5)	38,260,640 (16.3)	19,080,935 (16.3)	2,805,636 (15.5)	1,887,592 (17.8)
Tuesday	155,565,333 (14.6)	98,022,792 (14.3)	36,189,001 (15.4)	17,381,428 (14.9)	2,593,571 (14.3)	1,378,541 (13.0)
Wednesday	152,566,716 (14.3)	98,207,296 (14.3)	33,715,222 (14.3)	16,745,407 (14.3)	2,444,785 (13.5)	1,454,007 (13.7)
Thursday	146,894,239 (13.8)	94,402,962 (13.8)	32,608,278 (13.9)	15,824,170 (13.5)	2,595,204 (14.3)	1,463,625 (13.8)
Friday	148,615,588 (13.9)	95,474,110 (13.9)	32,379,118 (13.8)	16,819,533 (14.4)	2,563,946 (14.1)	1,378,881 (13.0)
Saturday	147,692,258 (13.9)	96,574,059 (14.1)	31,521,334 (13.4)	15,529,347 (13.3)	2,583,366 (14.3)	1,484,153 (14.0)
**Arrive by ambulance**	196,390,765 (18.8)	131,522,933 (19.6)	41,232,649 (17.9)	17,981,146 (15.8)	3,507,756 (19.8)	2,146,282 (20.7)
**Seen within last 72 h**	44,700,888 (4.8)	29,205,272 (4.8)	9,031,747 (4.5)	5,039,648 (4.9)	914,018 (5.5)	510,202 (5.2)
**Pain level**
No pain	191,922,708 (23.1)	126,398,959 (23.4)	40,018,910 (22.1)	19,382,697 (21.7)	4,284,336 (30.1)	1,837,805 (21.6)
Mild	90,787,924 (10.9)	63,142,483 (11.7)	15,962,562 (8.8)	9,413,458 (10.6)	1,737,260 (12.2)	532,160 (6.3)
Moderate	255,116,768 (30.6)	165,947,253 (30.8)	53,107,523 (29.3)	28,833,991 (32.3)	4,724,159 (33.2)	2,503,842 (29.4)
Severe	294,597,482 (35.4)	183,760,861 (34.1)	72,162,722 (39.8)	31,540,073 (35.4)	3,503,778 (24.6)	3,630,048 (42.7)
**Temperature**						
36–38°C	923,981,783 (92.0)	590,014,678 (91.6)	207,627,018 (93.4)	101,661,293 (92.1)	15,433,830 (90.9)	9,244,962 (92.3)
≤ 36°C	58,681,432 (5.8)	41,248,048 (6.4)	10,024,208 (4.5)	5,920,677 (5.4)	977,390 (5.8)	511,109 (5.1)
≥ 38°C	21,147,842 (2.1)	12,837,322 (2.0)	4,696,475 (2.1)	2,778,455 (2.5)	570,379 (3.4)	265,210 (2.6)
**Heart rate**						
≤ 90	692,407,280 (65.0)	437,892,700 (63.9)	155,914,279 (66.2)	79,870,849 (68.3)	12,105,387 (66.8)	6,624,066 (62.4)
90–100	177,024,146 (16.6)	114,148,145 (16.7)	40,367,221 (17.2)	17,916,420 (15.3)	2,787,718 (15.4)	1,804,643 (17.0)
100–110	100,961,893 (9.5)	68,101,115 (9.9)	20,940,376 (8.9)	9,327,277 (8.0)	1,618,249 (8.9)	974,876 (9.2)
110–120	51,685,009 (4.8)	35,362,040 (5.2)	10,098,936 (4.3)	4,775,100 (4.1)	753,308 (4.2)	695,625 (6.6)
> 120	43,858,506 (4.1)	29,444,993 (4.3)	8,036,068 (3.4)	5,004,119 (4.3)	858,354 (4.7)	514,971 (4.9)
**DBP**						
<60	490,634,962 (46.0)	315,052,788 (46.0)	104,018,788 (44.2)	57,632,101 (49.3)	9,111,084 (50.3)	4,820,201 (45.4)
60–80	103,631,700 (9.7)	68,712,622 (10.0)	20,434,534 (8.7)	11,619,767 (9.9)	1,818,276 (10.0)	1,046,501 (9.9)
> 80	471,670,172 (44.2)	301,183,584 (44.0)	110,903,557 (47.1)	47,641,896 (40.8)	7,193,657 (39.7)	4,747,479 (44.7)
**Census Region**						
Northeast	191,192,054 (17.9)	129,999,216 (19.0)	29,802,911 (12.7)	27,427,725 (23.5)	3,310,281 (18.3)	651,921 (6.1)
Midwest	250,844,979 (23.5)	178,113,941 (26.0)	58,620,522 (24.9)	10,411,755 (8.9)	2,115,228 (11.7)	1,583,533 (14.9)
South	421,782,783 (39.6)	251,090,677 (36.7)	130,502,027 (55.4)	35,832,957 (30.7)	2,816,720 (15.5)	1,540,402 (14.5)
West	202,117,020 (19.0)	125,745,159 (18.4)	16,431,418 (7.0)	43,221,328 (37.0)	9,880,788 (54.5)	6,838,327 (64.4)
**Reason for visit**						
General symptoms	203,213,691 (19.1)	131,873,758 (19.3)	44,378,354 (18.9)	21,275,654 (18.3)	3,922,060 (21.7)	1,763,865 (16.7)
Symptoms referable to psychological and mental disorders	32,995,307 (3.1)	22,552,772 (3.3)	6,386,028 (2.7)	3,255,770 (2.8)	504,223 (2.8)	296,513 (2.8)
Symptoms referable to the nervous system	83,266,087 (7.8)	53,334,813 (7.8)	18,462,744 (7.9)	9,206,729 (7.9)	1,485,478 (8.2)	776,323 (7.3)
Symptoms referable to the cardiovascular and lymphatic systems	21,182,499 (2.0)	13,986,368 (2.0)	4,764,015 (2.0)	1,887,719 (1.6)	435,322 (2.4)	109,075 (1.0)
Symptoms referable to the eyes and ears	23,544,404 (2.2)	14,145,130 (2.1)	5,806,456 (2.5)	2,924,836 (2.5)	471,989 (2.6)	195,993 (1.9)
Symptoms referable to the respiratory system	108,894,097 (10.2)	68,165,421 (10.0)	27,305,739 (11.6)	10,599,465 (9.1)	1,704,670 (9.4)	1,118,803 (10.6)
Symptoms referable to the digestive system	166,981,035 (15.7)	103,946,767 (15.2)	36,765,578 (15.7)	21,423,846 (18.4)	2,833,010 (15.7)	2,011,834 (19.0)
Symptoms referable to the genitourinary system	54,443,113 (5.1)	29,878,987 (4.4)	15,254,564 (6.5)	7,710,106 (6.6)	1,140,625 (6.3)	458,830 (4.3)
Symptoms referable to the skin, nails, and hair	34,639,996 (3.3)	21,236,239 (3.1)	8,272,821 (3.5)	4,098,009 (3.5)	710,716 (3.9)	322,212 (3.0)
Symptoms referable to the musculoskeletal system	169,150,947 (15.9)	109,127,670 (16.0)	38,665,017 (16.5)	17,543,747 (15.1)	2,089,632 (11.6)	1,724,881 (16.3)
Other	164,108,043 (15.4)	114,520,152 (16.8)	28,509,476 (12.2)	16,496,810 (14.2)	2,771,491 (15.3)	1,810,115 (17.1)

*The missing proportion for arrival time, residency type, insurance, and residency type is <5%; the missing proportion for temperature, heart rate, and blood pressure is 5–10%; the missing proportion for triage level is 16%, for Seen within last 72 h is 13%, for pain level is 21%*.

Tables 2, 3, and Supplement Table 3 summarized the main outcomes of interest across the sample as a whole and stratified by race/ethnicity. After adjusting for other covariates, black patients were 10% less likely (aOR 0.93, 95% CI 0.90–0.97) to receive immediate or emergent as opposed to semi- or non-urgent scores. Similarly, Hispanic patients were 8% more likely (aOR 1.08, 95% CI 1.03–1.13) than white patients to receive immediate or emergent scores as opposed to semi- or non-urgent scores. Asian ED patients were more likely than white patients to receive immediate and emergent (aOR 1.19, 95% CI 1.08–1.30) or urgent care (aOR 1.13, 95% CI 1.05–1.21) scores as opposed to semi- or non-urgent care needs in all models.

**Table 2:** Proportion of emergency severity index, hospital admission, ICU admission, medical resources utilization, stratified by race/ethnicities, NHAMCS 2005–2016 (weighted sample).

	**All**	**White patients**	**Black patients**	**Hispanic patients**	**Asian patients**	**Other**
**ESI score**						
1–Immediate	24,176,650 (2.7)	16,835,752 (2.9)	4,760,815 (2.5)	2,004,310 (2.1)	362,564 (2.4)	213,209 (2.5)
2–Emergent	111,093,774 (12.6)	75,525,674 (13.2)	22,039,245 (11.4)	10,341,490 (10.8)	2,232,668 (14.5)	954,698 (11.3)
3–Urgent	417,130,120 (47.1)	271,814,776 (47.5)	87,648,287 (45.4)	45,935,756 (47.9)	7,768,313 (50.5)	3,962,988 (46.8)
4–Semi-urgent	262,314,439 (29.6)	165,371,860 (28.9)	61,097,051 (31.7)	28,975,364 (30.2)	4,182,829 (27.2)	2,687,335 (31.7)
5–Non-urgent	70,396,103 (8.0)	42,986,980 (7.5)	17,331,203 (9.0)	8,580,884 (9.0)	844,076 (5.5)	652,960 (7.7)
Hospital Admission	171,492,659 (16.1)	121,926,052 (17.8)	30,310,443 (12.9)	14,290,598 (12.2)	3,460,811 (19.1)	1,504,755 (14.2)
ICU	20,678,842 (1.9)	14,678,517 (2.1)	3,830,975 (1.6)	1,578,750 (1.4)	392,470 (2.2)	198,131 (1.9)
ED/In-hospital death	5,797,774 (0.5)	4,160,464 (0.6)	1,067,849 (0.5)	365,729 (0.3)	175,796 (1.0)	27,936 (0.3)
Blood test	512,300,921 (48.1)	336,577,728 (49.1)	105,873,762 (45.0)	55,383,422 (47.4)	9,732,635 (53.7)	4,733,374 (44.6)
Any image	538,613,213 (50.5)	361,948,731 (52.8)	106,748,828 (45.4)	55,835,981 (47.8)	9,284,380 (51.2)	4,795,295 (45.2)
Procedure	527,191,203 (49.5)	344,838,866 (50.3)	109,011,311 (46.3)	58,116,519 (49.7)	10,160,146 (56.1)	5,064,363 (47.7)
Waiting time (min, MEANS (95% CI))	49.7 (49.4–50.0)	45.2 (44.8–45.6)	60.3 (59.5–61.1)	55.3 (54.2–56.4)	49.9 (47.5–52.2)	45.8 (42.8–48.9)
Length of visit (min, MEANS (95% CI))	222.0 (221.0–223.0)	211.8 (210.5–213.0)	237.7 (235.4–239.9)	249.5 (245.8–253.2)	243.3 (235.9–250.7)	201.1 (192.4–209.8)

*Waiting time, time from arrival to seeing the physician; Length of visit, time from arrival to discharge*.

**Table 3:** Odds ratio of emergency severity index, hospital admission, ICU admission, medical resources utilization, stratified by race/ethnicity, NHAMCS 2005–2016.

	**Racial/ethnic group**	**Crude odds ratio**	Adjusted for^*^			
			**Demographics**	**+ Socioeconomic**	**+ Visiting & clinical**	**+ESI scores**
**ESI score: Immediate or emergent vs. semi- or non-urgent**	**White**	**(****1****)**	**(****1****)**	**(****1****)**	**(****1****)**	
	Black	0.82 (0.79–0.84)	0.95 (0.92–0.98)	0.98 (0.95–1.01)	0.93 (0.90–0.97)	
	Hispanic	0.88 (0.85–0.92)	1.05 (1.01–1.10)	1.07 (1.02–1.12)	1.08 (1.03–1.13)	
	Asian	1.23 (1.13–1.34)	1.24 (1.13–1.35)	1.23 (1.13–1.34)	1.19 (1.08–1.30)	
	Other	0.76 (0.66–0.87)	0.85 (0.74–0.97)	0.85 (0.74–0.97)	0.80 (0.69–0.93)	
**ESI score: Urgent vs. semi- or non-urgent**	**White**	**(****1****)**	**(****1****)**	**(****1****)**	**(****1****)**	
	Black	0.90 (0.88–0.92)	0.98 (0.95–1.00)	1.01 (0.98–1.03)	0.98 (0.95–1.00)	
	Hispanic	1.00 (0.97–1.03)	1.11 (1.07–1.14)	1.11 (1.08–1.15)	1.08 (1.05–1.12)	
	Asian	1.22 (1.14–1.30)	1.22 (1.15–1.31)	1.17 (1.10–1.25)	1.13 (1.05–1.21)	
	Other	0.98 (0.89–1.07)	1.04 (0.95–1.14)	1.00 (0.91–1.10)	0.95 (0.86–1.04)	
**Hospital Admission**	**White**	**(****1****)**	**(****1****)**	**(****1****)**	**(****1****)**	
	Black	0.73 (0.71–0.75)	0.94 (0.91–0.97)	0.93 (0.91–0.96)	0.90 (0.87–0.93)	0.90 (0.87–0.93)
	Hispanic	0.72 (0.69–0.74)	0.94 (0.91–0.98)	0.97 (0.94–1.01)	0.97 (0.93–1.01)	1.01 (0.97–1.06)
	Asian	1.17 (1.10–1.25)	1.17 (1.10–1.25)	1.28 (1.20–1.38)	1.23 (1.14–1.32)	1.21 (1.12–1.31)
	Other	0.82 (0.74–0.92)	1.00 (0.89–1.12)	1.09 (0.97–1.22)	1.00 (0.89–1.13)	0.98 (0.86–1.13)
**ICU**	**White**	**(****1****)**	**(****1****)**	**(****1****)**	**(****1****)**	
	Black	0.81 (0.75–0.87)	1.11 (1.03–1.19)	1.10 (1.02–1.19)	1.09 (1.00–1.18)	1.14 (1.05–1.24)
	Hispanic	0.65 (0.59–0.72)	0.90 (0.81–1.00)	0.91 (0.82–1.01)	0.96 (0.86–1.07)	0.97 (0.86–1.09)
	Asian	0.96 (0.80–1.15)	0.94 (0.78–1.13)	0.96 (0.80–1.16)	0.95 (0.78–1.15)	0.92 (0.75–1.14)
	Other	0.83 (0.62–1.11)	1.03 (0.77–1.39)	1.07 (0.79–1.44)	0.96 (0.70–1.30)	0.93 (0.66–1.31)
**Death**	**White**	**(****1****)**	**(****1****)**	**(****1****)**	**(****1****)**	
	Black	0.74 (0.65–0.86)	1.22 (1.05–1.40)	1.18 (1.02–1.37)	1.23 (1.05–1.44)	1.26 (1.06–1.49)
	Hispanic	0.58 (0.47–0.71)	0.93 (0.76–1.15)	0.96 (0.78–1.18)	1.05 (0.84–1.30)	1.12 (0.88–1.42)
	Asian	1.49 (1.13–1.96)	1.45 (1.10–1.91)	1.53 (1.15–2.05)	1.64 (1.20–2.22)	1.64 (1.17–2.31)
	Other	0.63 (0.34–1.17)	0.88 (0.47–1.65)	0.92 (0.49–1.73)	0.71 (0.36–1.41)	0.82 (0.40–1.66)
**Blood test**	**White**	**(****1****)**	**(****1****)**	**(****1****)**	**(****1****)**	
	Black	0.87 (0.85–0.89)	1.01 (0.99–1.03)	1.00 (0.98–1.02)	0.94 (0.92–0.96)	0.96 (0.94–0.99)
	Hispanic	0.96 (0.93–0.98)	1.14 (1.11–1.17)	1.17 (1.14–1.20)	1.13 (1.10–1.16)	1.15 (1.11–1.19)
	Asian	1.24 (1.17–1.30)	1.24 (1.18–1.31)	1.28 (1.22–1.36)	1.20 (1.13–1.28)	1.22 (1.14–1.31)
	Other	0.95 (0.88–1.03)	1.07 (0.99–1.16)	1.11 (1.02–1.20)	1.02 (0.94–1.12)	0.96 (0.86–1.06)
**Any Imaging**	**White**	**(****1****)**	**(****1****)**	**(****1****)**	**(****1****)**	
	Black	0.73 (0.72–0.75)	0.83 (0.82–0.85)	0.84 (0.83–0.86)	0.83 (0.81–0.85)	0.84 (0.82–0.86)
	Hispanic	0.79 (0.77–0.81)	0.91 (0.89–0.94)	0.98 (0.96–1.01)	1.02 (0.99–1.05)	1.04 (1.01–1.07)
	Asian	0.97 (0.92–1.02)	0.96 (0.91–1.02)	1.05 (0.99–1.10)	1.15 (1.08–1.21)	1.16 (1.09–1.23)
	Other	0.81 (0.75–0.88)	0.90 (0.83–0.97)	0.99 (0.92–1.08)	0.96 (0.88–1.04)	0.98 (0.89–1.07)
**Procedure**	**White**	**(****1****)**	**(****1****)**	**(****1****)**	**(****1****)**	
	Black	0.85 (0.83–0.86)	0.91 (0.89–0.93)	0.94 (0.92–0.96)	0.95 (0.93–0.97)	0.96 (0.94–0.98)
	Hispanic	1.00 (0.98–1.03)	1.08 (1.05–1.11)	1.10 (1.07–1.13)	1.10 (1.07–1.13)	1.10 (1.07–1.13)
	Asian	1.21 (1.15–1.27)	1.20 (1.14–1.27)	1.16 (1.10–1.22)	1.16 (1.10–1.22)	1.15 (1.08–1.21)
	Other	0.95 (0.88–1.02)	1.00 (0.93–1.08)	0.98 (0.90–1.05)	0.95 (0.87–1.02)	0.93 (0.86–1.02)

* *+ Demographics adjusts for gender, age group; + Socioeconomic adjusts for residence type, insurance type, census region; + Visiting & clinical: year, week of day, arrive by ambulance, seen within last 72 h, pain level, temperature, heart rate, dialytic blood pressure*.

After adjusting for other covariates (including ESI level), black patients and Hispanic patients were also 10% less likely than white patients to be admitted to the hospital following their ED visit (aOR 0.90, CI 0.87–0.93). Asian patients were 1.21 times more likely than white patients to be admitted to the hospital following an ED visit (aOR 1.21, 95% CI 1.12–1.31). Black patients were 1.14 times more likely to receive ICU admission in the fully adjusted models (aOR 1.14, 95% CI: 1.05–1.24). Odds of dying in the hospital/ED differed among the groups. Relative to white patients, Black patients were 1.26 times more likely to die in hospital (aOR 1.26, 95% CI 1.06–1.49); Hispanic patients, 1.12 times more likely (aOR 1.10, 95% CI 0.88–1.42); Asian patients, 1.64 (aOR 1.64, 95% CI 1.17–2.31).

After adjusting for other covariates (including ESI level), black patients were 16% (aOR 0.84, 95% CI 0.82–0.86) less likely to receive any imaging and 4% (aOR 0.96, 95% CI 0.94–0.99) less likely to have a blood test during ED visit than white patients. In contrast, Asian patients were 1.22 (aOR 1.22, 95% CI 1.14 −1.31) times more likely than white patients to receive a blood test. Hispanic and Asian patients were 1.10 times and 1.15 times (aOR 1.12, 95% CI 1.07–1.13; aOR 1.15, 95% CI 1.08–1.21) more likely to receive a procedure in the ED than white patients. After adjusting for other covariates, waiting times in the ED were significantly longer for all minorities (*p* < 0.001) as compared to white patients (Table 4).

**Table 4:** Linear regression between log transform of wait time or length of visit and by race/ethnicity, NHAMCS 2005–2016.

	**Wait time**		**Length of visit**	
	**Beta (95% CI)**	***p*-value**	**Beta (95% CI)**	***p*-value**
White	(1)		(1)	
Black	0.27 (0.25–0.28)	<0.0001	0.18 (0.17–0.19)	<0.0001
Hispanic	0.13 (0.11–0.15)	<0.0001	0.21 (0.20–0.22)	<0.0001
Asian	0.06 (0.02–0.09)	0.001	0.13 (0.11–0.16)	<0.0001
Other	0.08 (0.03–0.13)	0.002	−0.01 (−0.04–0.02)	0.45

*The model was adjusted for the following factors: demographics (gender, age group), socioeconomic (residence type, insurance type, census region), visiting and clinical (year, week of day, arrive by ambulance, seen within last 72 h, pain level, temperature, heart rate, dialytic blood pressure), and ESI score*.

**Figure 1:**
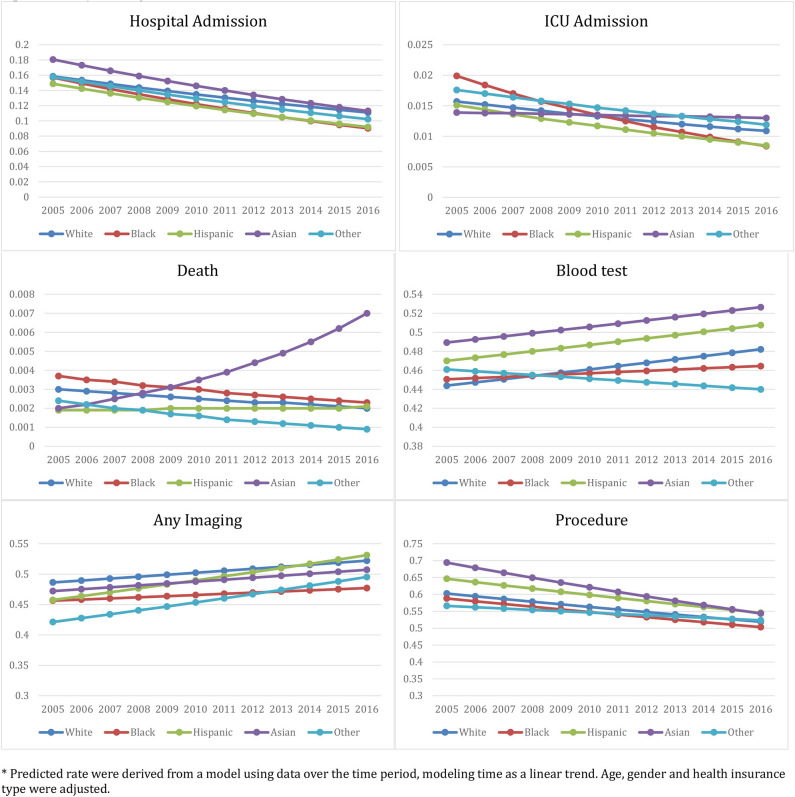
Racial/Ethnic-Specific ED health outcome and medical resource utilization rate from 2005 to 2016: NHAMCS 2005–2016. *Predicted rate were derived from a model using data over the time period, modeling time as a linear trend. Age, gender, and health insurance type were adjusted.

Figure 1 displays trends of different health outcome and resource utilization variables over time (2006–2016) by racial/ethnic group. Table 5 includes the estimated health outcome and resource utilization rates and changes over time. Rates of hospital and ICU admission significantly decreased over time in all racial/ethnic groups. However, these rates decreased the least in white patients as compared to other groups (*p* < 0.01). Of particular note, hospital and ICU admission for white patients decreased by 30.01 and 30.57%, respectively, compared to 42.48 and 57.79% for black patients. Death rates in the ED for black and white patients decreased over the 12 years by 37.84 and 33.33%, respectively, whereas the Asian and Hispanic patients' death rates increased by 250.00 and 10.53%, respectively (see Discussion for analysis of the large increase for Asian patients). Blood test rates and medical imaging utilization rates in the ED increased (though with different 2006–2016 percent changes) while the procedure utilization rates dropped across all racial/ethnic groups.

**Table 5:** Race/ethnicity-specific rates and trends of health outcome and medical resources utilization: NHAMCS 2005–2016.

		Rate^*^		**Trend**	***p*-value**	***p***^†^****
**Outcome**	**Race/ethnicity**	**2005**	**2016**	**2005–2016**	**trend**	**Racial/ethnic difference in trend**
Hospital Admission	White	0.159	0.111	−30.01%	<0.001	Reference
	Black	0.157	0.090	−42.48%	<0.001	<0.001
	Hispanic	0.149	0.092	−38.10%	<0.001	<0.001
	Asian	0.181	0.113	−37.41%	<0.001	<0.001
	Other	0.158	0.102	−35.09%	<0.001	<0.001
ICU Admission	White	0.016	0.011	−30.57%	<0.001	Reference
	Black	0.020	0.008	−57.79%	<0.001	<0.001
	Hispanic	0.015	0.009	−43.71%	<0.001	<0.001
	Asian	0.014	0.013	−6.47%	<0.001	<0.001
	Other	0.018	0.012	−32.39%	<0.001	0.0035
Death	White	0.003	0.002	−33.33%	<0.001	Reference
	Black	0.004	0.002	−37.84%	<0.001	<0.001
	Hispanic	0.002	0.002	10.53%	<0.001	<0.001
	Asian	0.002	0.007	250.00%	<0.001	<0.001
	Other	0.002	0.001	−62.50%	<0.001	<0.001
Blood test	White	0.444	0.482	8.61%	<0.001	Reference
	Black	0.451	0.465	3.11%	<0.001	<0.001
	Hispanic	0.470	0.508	8.02%	<0.001	<0.001
	Asian	0.489	0.526	7.60%	<0.001	<0.001
	Other	0.461	0.440	−4.53%	<0.001	<0.001
Any imaging	White	0.486	0.522	7.36%	<0.001	Reference
	Black	0.456	0.477	4.54%	<0.001	<0.001
	Hispanic	0.458	0.531	16.11%	<0.001	<0.001
	Asian	0.472	0.507	7.41%	<0.001	<0.001
	Other	0.421	0.495	17.59%	<0.001	<0.001
Procedure	White	0.603	0.519	−13.87%	<0.001	Reference
	Black	0.588	0.503	−14.41%	<0.001	<0.001
	Hispanic	0.646	0.546	−15.59%	<0.001	<0.001
	Asian	0.694	0.543	−21.71%	<0.001	<0.001
	Other	0.566	0.524	−7.53%	<0.001	<0.001

* *Predicted rate and trend were derived from a model using data over the time period, modeling time as a linear trend*.

† *From the time by Race/ethnicity interaction in the Poisson regression model*.

## Discussion

We observed significant racial/ethnic differences in the evaluation and management of adult patients in the ED based on the nationally representative NHAMCS, between 2005 and 2016. In the ED, black patients and those in the other racial/ethnic group were less likely than white patients to receive immediate or urgent ESI scores as opposed to semi- or non-urgent care needs. Our study indicates that this racial/ethnic disparity could not be explained by demographic, socioeconomic, or factors related to the patients' clinical presentation or the context of their visit. While we cannot infer the cause of these ESI disparities based on the current study, we point to two potential explanations. First, as past research has found, racial/ethnic minorities are less likely than white patients to have a primary care provider and are more likely to rely on ED services for routine care needs (2–4). This differential use of the healthcare system by racial groups may partially account for the disparate ESI scores. Alternatively, racial bias on the part of nurses and other healthcare providers may contribute to the lower ESI scores assigned to racial and ethnic minorities. The influence of racial bias in ED decision-making has been noted in prior research (20), including one study of a large, urban-based university hospital in the US (27), but further observational research is needed to better understand the complex interplay of healthcare utilization patterns and the patient experience of different racial/ethnic groups.

Unlike the other racial/ethnic minority groups, Asian patients were more likely than white patients to present to the ED with immediate or urgent care needs. While the higher socioeconomic indicators ascribed to the Asian patients in our sample (e.g., higher rates of private insurance and inhabiting a private residence) may contribute, it is worth noting that the pattern remained in the fully adjusted models accounting for these factors. Unfortunately, due to a lack of granularity in the NHAMCS-ED, we were unable to further stratify the Asian sample by region or country of origin, nor could we test for the effects of patient-level factors such as income, education, primary language, or duration of US residence on the outcomes analyzed in this study. Such information would be valuable in attempting to explain the divergent patterns observed between Asian patients and other racial/ethnic minorities included in our sample (28).

Black groups were also less likely than white patients to be admitted to the hospital following an ED encounter after controlling for model covariates. Compared to white patients, Asian patients were either more or equally likely to be admitted to the hospital following an ED visit, which again diverges from the pattern of the other racial/ethnic groups and cannot be fully explained by the factors available in the NHAMCS-ED dataset. Although mortality rates were <1% for all groups, Asian and black patients were more likely than white patients to experience in-hospital death following an ED visit, after we adjusted for the patients' ESI scores. While the data indicate that Asian patients have remarkably high odds (1.93), compared to white patients, of dying in the ED/hospital, we suspect that this result may owe in part to the small sample size of Asian patients relative to the other racial/ethnic groups and the infrequency of deaths in the ED/hospital (Supplement Table 3, unweighted *N* = 54).

In the adjusted models, we also found that black and other racial/ethnic patients were less likely than white patients to receive blood tests or other procedures in their ED visit. In contrast, Asian and Hispanic patients were more likely than white patients to receive blood tests or other procedures in the ED. The persistence of these disparities after stringent adjustment in our models suggests a need for further observational research within EDs. For example, research is needed to understand why Hispanic patients, who fared worse than white patients on most outcomes in our analysis, have higher utilization rates for blood tests and other procedures relative to white patients. It is particularly noteworthy that adjusting for ESI score did not eliminate racial/ethnic disparities in hospital/ICU admission, death in the ED/hospital, or medical resource utilization. This finding indicates that, even if racial/ethnic bias does not influence ESI score assignment, such biases may still influence healthcare providers' decision-making throughout the emergency care process.

We further found that black patients and Hispanic patients endured significantly longer wait times as compared to white patients, even after adjusting for ESI scores. This finding is consistent with prior research pointing to the demographic case mix and volume of visits within individual emergency departments as primary contributing factors to differential wait times among racial/ethnic groups (29, 30). However, all three of the main racial/ethnic minority groups in our analysis were found to spend more time in the ED overall, with the longest visits experienced by Hispanic patients. It is possible that a need for language translation may partially contribute to this outcome (31, 32), but we were not able to control for primary language or use of translation services in our analysis.

Finally, our modeling of trends from 2005 to 2016 suggests that certain outcomes in ED care were improving across all racial/ethnic groups at a consistent rate. However, racial/ethnic minorities' rate of improvement lagged behind that of white patients in terms of ICU admission rates, risk of death in the ED/hospital, and levels of general procedures and blood tests performed during ED visits. The largest disparity existed between black and white patients over this 12-year period. Future clinical interventions and health policies should focus on targeting improvements toward racial and ethnic minorities, especially black and Hispanic patients.

## Limitations

The major limitation of our study relates to potential sampling biases and errors inherent in the NHAMCS-ED data. Namely, heterogeneity in documentation (e.g., due to differences in electronic health records practices) may involve abstraction errors, missing responses, and inaccurate responses. However, we do not suspect that such systematic biases would moderate the associations with race/ethnicity noted herein in any consistent way. Additionally, 6% of the total NHAMCS-ED sample was removed for not having a documented race/ethnicity; however, this figure is below the acceptable non-response threshold for this type of data source and thus does not invalidate the primary exposure variable for this analysis (33). Another limitation of our study is our use of broader system-based reasons for ED visit (e.g., “symptoms referable to the respiratory system”) as a unit of analysis in our model, rather than performing the more limited analysis that would result from examining each chief complaint (e.g., “shortness of breath”). Future investigations into disparities in ED outcomes and resource utilization for more specific reasons for visit could be revealing.

## Conclusions

The emergency care of black patients was characterized by disparities in multiple dimensions of care. Namely, black patients received lower ESI scores, were less likely to receive tests in the ED, were less likely to be admitted to the hospital and/or ICU, and had a higher death rate in the ED and hospital. Some of these findings were in contrast to Hispanic and Asian patients, who, in general, received equivalent or greater ED resources compared to white patients. Further research is needed to understand the underlying causes and long-term health consequences of these racial/ethnic disparities in ED care in order to inform clinical guidelines and policies for eliminating racial differences in this critical area of US healthcare.

## Data Availability Statement

Publicly available datasets were analyzed in this study. The data can be accessed from the CDC website: https://www.cdc.gov/nchs/ahcd/index.htm.

## Author Contributions

XZ: full access to all the data in the study and takes responsibility for the integrity of the data and accuracy of the data analysis. XZ and PM: concept and design. MC, XZ, and TH: drafting of the manuscript. RS, SB, and PM: critical revision of the manuscript for important intellectual content. XZ: statistical analysis. XZ and PM: obtained funding. XZ, MC, and TH: administrative, technical, or material support. XZ and PM: supervision. All authors: acquisition, analysis, or interpretation of data.

## Conflict of Interest

The authors declare that the research was conducted in the absence of any commercial or financial relationships that could be construed as a potential conflict of interest.
