# Supplementary material for: Trends of Racial/Ethnic Differences in Emergency Department Care Outcomes Among Adults in the United States From 2005 to 2016
Source: Front Med (Lausanne). 2020 Jun 25;7:300. doi: 10.3389/fmed.2020.00300 (PMC7330111; doi:10.3389/fmed.2020.00300)
Supplement: Supplementary file 1 [file Table_1.docx]

Supplement Table 1. Types of Procedures Collected in the NHAMCS 2005–2016

| Year | Procedure |
| --- | --- |
| 2012–2016 | BiPAP/CPAP; Bladder catheter; Cast, splint, wrap; Central line Other; IV fluids; CPR; Endotracheal intubation; Incision & drainage (I&D); IV fluids; Lumbar puncture (LP); Nebulizer therapy; Pelvic exam; Skin adhesives; Suturing/Staples; Other |
| 2007–2011 | IV fluids; Cast; splint or wrap; Suturing/Staples; Incision & drainage (I&D); Foreign body removal; Nebulizer therapy; Bladder catheter; Pelvic exam; Central line; CPR; Endotracheal intubation; Other |
| 2005–2006 | Bladder catheter; CPR; Endotracheal intubation; IV fluids; Nebulizer therapy; NG tube/gastric suction; OB/GYN care; orthopedic care; Thrombolytic therapy; Wound care; Other |

Supplement Table 2. Baseline Characteristics of Patients Presenting to the ED, Stratified by Race/Ethnicity, NHAMCS 2005–2016 (Unweighted Sample)

|  | All | White patients | Black patients | Hispanic patients | Asian patients | Other |
| --- | --- | --- | --- | --- | --- | --- |
|  | 247,989 | 155918 (62.9) | 54451 (22.0) | 29106 (11.7) | 5918 (2.4) | 2596 (1.1) |
| **Male** | 108,231(43.6) | 69,069(44.3) | 22,695(41.7) | 12,707(43.7) | 2,623(44.3) | 1,137(43.8) |
| **Age** |  |  |  |  |  |  |
| 18–39 | 105,943(42.7) | 60,663(38.9) | 26,296(48.3) | 15,436(53.0) | 2,333(39.4) | 1,215(46.8) |
| 40–49 | 42,982(17.3) | 25,854(16.6) | 10,671(19.6) | 5,131(17.6) | 869(14.7) | 457(17.6) |
| 50–59 | 36,646(14.8) | 22,911(14.7) | 8,706(16.0) | 3,738(12.8) | 887(15.0) | 404(15.6) |
| 60–74 | 33,715(13.6) | 23,568(15.1) | 5,878(10.8) | 2,983(10.2) | 939(15.9) | 347(13.4) |
| ≥ 75 | 28,703(11.6) | 22,922(14.7) | 2,900(5.3) | 1,818(6.2) | 890(15.0) | 173(6.7) |
| **Residence type** |  |  |  |  |  |  |
| Private residence | 225,574(94.6) | 141,363(94.2) | 49,857(94.9) | 26,500(95.5) | 5,456(95.9) | 2,398(95.1) |
| Nursing home | 6,038(2.5) | 4,689(3.1) | 880(1.7) | 314(1.1) | 129(2.3) | 26(1.0) |
| Homeless | 2,577(1.1) | 1,332(0.9) | 766(1.5) | 391(1.4) | 33(0.6) | 55(2.2) |
| Other | 4,335(1.8) | 2,653(1.8) | 1,031(2.0) | 535(1.9) | 73(1.3) | 43(1.7) |
| **Insurance type** |  |  |  |  |  |  |
| Private insurance | 74,338(31.8) | 52,842(35.6) | 12,190(24.0) | 6,393(23.9) | 2,179(39.5) | 734(29.4) |
| Medicare | 54,915(23.5) | 41,428(27.9) | 8,593(16.9) | 3,387(12.7) | 1,073(19.5) | 434(17.4) |
| Medicaid or CHIP | 53,100(22.7) | 25,785(17.4) | 16,479(32.4) | 8,774(32.9) | 1,305(23.7) | 757(30.3) |
| Uninsured | 41,315(17.7) | 22,119(14.9) | 11,599(22.8) | 6,500(24.3) | 684(12.4) | 413(16.5) |
| Other | 10,249(4.4) | 6,197(4.2) | 1,970(3.9) | 1,648(6.2) | 272(4.9) | 162(6.5) |
| **Year** |  |  |  |  |  |  |
| 2005 | 24,448(9.9) | 15,353(9.8) | 4,935(9.1) | 3,251(11.2) | 661(11.2) | 248(9.6) |
| 2006 | 25,891(10.4) | 15,934(10.2) | 5,612(10.3) | 3,265(11.2) | 799(13.5) | 281(10.8) |
| 2007 | 24,310(9.8) | 15,249(9.8) | 5,664(10.4) | 2,602(8.9) | 585(9.9) | 210(8.1) |
| 2008 | 22,761(9.2) | 14,441(9.3) | 5,328(9.8) | 2,281(7.8) | 494(8.3) | 217(8.4) |
| 2009 | 24,309(9.8) | 15,090(9.7) | 5,718(10.5) | 2,653(9.1) | 596(10.1) | 252(9.7) |
| 2010 | 24,757(10.0) | 15,643(10.0) | 5,359(9.8) | 2,848(9.8) | 617(10.4) | 290(11.2) |
| 2011 | 21,884(8.8) | 13,801(8.9) | 4,601(8.4) | 2,548(8.8) | 611(10.3) | 323(12.4) |
| 2012 | 20,247(8.2) | 12,836(8.2) | 4,108(7.5) | 2,683(9.2) | 407(6.9) | 213(8.2) |
| 2013 | 16,550(6.7) | 10,320(6.6) | 3,919(7.2) | 1,823(6.3) | 344(5.8) | 144(5.5) |
| 2014 | 15,319(6.2) | 9,593(6.2) | 3,390(6.2) | 1,916(6.6) | 277(4.7) | 143(5.5) |
| 2015 | 14,041(5.7) | 9,079(5.8) | 2,954(5.4) | 1,688(5.8) | 218(3.7) | 102(3.9) |
| 2016 | 13,472(5.4) | 8,579(5.5) | 2,863(5.3) | 1,548(5.3) | 309(5.2) | 173(6.7) |
| **Day of Week** |  |  |  |  |  |  |
| Sunday | 34,262(13.8) | 22,131(14.2) | 7,148(13.1) | 3,774(13.0) | 828(14.0) | 381(14.7) |
| Monday | 38,924(15.7) | 24,123(15.5) | 8,684(15.9) | 4,757(16.3) | 938(15.8) | 422(16.3) |
| Tuesday | 36,210(14.6) | 22,297(14.3) | 8,345(15.3) | 4,329(14.9) | 854(14.4) | 385(14.8) |
| Wednesday | 35,451(14.3) | 22,117(14.2) | 7,944(14.6) | 4,247(14.6) | 795(13.4) | 348(13.4) |
| Thursday | 34,308(13.8) | 21,526(13.8) | 7,604(14.0) | 3,995(13.7) | 838(14.2) | 345(13.3) |
| Friday | 34,681(14.0) | 21,822(14.0) | 7,568(13.9) | 4,115(14.1) | 813(13.7) | 363(14.0) |
| Saturday | 34,153(13.8) | 21,902(14.0) | 7,158(13.1) | 3,889(13.4) | 852(14.4) | 352(13.6) |
| **Arrive by ambulance** | 46,444(19.2) | 29,909(19.6) | 10,031(18.9) | 4,806(17.0) | 1,193(20.6) | 505(19.8) |
| **Seen within last 72 hours** | 10,548(4.9) | 6,625(4.8) | 2,078(4.5) | 1,443(5.8) | 259(4.7) | 143(6.0) |
| **Pain level** |  |  |  |  |  |  |
| No pain | 47,744(24.5) | 30,114(24.4) | 10,177(23.9) | 5,307(23.9) | 1,607(33.5) | 539(25.3) |
| Mild | 22,941(11.8) | 15,290(12.4) | 4,130(9.7) | 2,696(12.1) | 667(13.9) | 158(7.4) |
| Moderate | 59,271(30.4) | 37,857(30.7) | 12,277(28.9) | 7,062(31.8) | 1,447(30.2) | 628(29.5) |
| Severe | 64,966(33.3) | 39,980(32.4) | 15,949(37.5) | 7,164(32.2) | 1,069(22.3) | 804(37.8) |
| **Temperature** |  |  |  |  |  |  |
| 36–38 °C | 212,428(91.3) | 133,033(90.9) | 47,136(92.2) | 24,968(91.4) | 5,060(90.8) | 2,231(91.2) |
| ≤ 36 °C | 15,204(6.5) | 10,203(7.0) | 2,867(5.6) | 1,670(6.1) | 329(5.9) | 135(5.5) |
| ≥ 38 °C | 5,126(2.2) | 3,072(2.1) | 1,112(2.2) | 680(2.5) | 181(3.2) | 81(3.3) |
| **Heart Rate** |  |  |  |  |  |  |
| ≤ 90 | 161,695(65.2) | 100,252(64.3) | 36,154(66.4) | 19,638(67.5) | 3,986(67.4) | 1,665(64.1) |
| 90–100 | 41,113(16.6) | 25,912(16.6) | 9,307(17.1) | 4,559(15.7) | 906(15.3) | 429(16.5) |
| 100–110 | 23,045(9.3) | 15,088(9.7) | 4,828(8.9) | 2,392(8.2) | 509(8.6) | 228(8.8) |
| 110–120 | 11,718(4.7) | 7,843(5.0) | 2,251(4.1) | 1,230(4.2) | 249(4.2) | 145(5.6) |
| > 120 | 10,418(4.2) | 6,823(4.4) | 1,911(3.5) | 1,287(4.4) | 268(4.5) | 129(5.0) |
| **DBP** |  |  |  |  |  |  |
| < 60 | 115,751(46.7) | 72,382(46.4) | 24,516(45.0) | 14,740(50.6) | 2,942(49.7) | 1,171(45.1) |
| 60–80 | 24,840(10.0) | 16,128(10.3) | 4,994(9.2) | 2,834(9.7) | 632(10.7) | 252(9.7) |
| > 80 | 107,398(43.3) | 67,408(43.2) | 24,941(45.8) | 11,532(39.6) | 2,344(39.6) | 1,173(45.2) |
| **US Census Region** |  |  |  |  |  |  |
| Northeast | 56,023(22.6) | 35,596(22.8) | 10,448(19.2) | 8,407(28.9) | 1,332(22.5) | 240(9.2) |
| Midwest | 55,882(22.5) | 40,155(25.8) | 12,217(22.4) | 2,801(9.6) | 440(7.4) | 269(10.4) |
| South | 87,939(35.5) | 51,471(33.0) | 27,470(50.4) | 8,000(27.5) | 586(9.9) | 412(15.9) |
| West | 48,145(19.4) | 28,696(18.4) | 4,316(7.9) | 9,898(34.0) | 3,560(60.2) | 1,675(64.5) |
| **Reason for visit** |  |  |  |  |  |  |
| General Symptoms | 47,409(19.2) | 29,740(19.1) | 10,556(19.5) | 5,406(18.7) | 1,209(20.5) | 498(19.3) |
| Symptoms Referable to Psychological and Mental Disorders | 9,454(3.8) | 5,729(3.7) | 2,136(3.9) | 1,256(4.3) | 246(4.2) | 87(3.4) |
| Symptoms Referable to the Nervous System | 19,133(7.7) | 12,184(7.8) | 4,106(7.6) | 2,157(7.5) | 513(8.7) | 173(6.7) |
| Symptoms Referable to the Cardiovascular and Lymphatic Systems | 4,665(1.9) | 3,088(2.0) | 999(1.8) | 431(1.5) | 115(2.0) | 32(1.2) |
| Symptoms Referable to the Eyes and Ears | 5,525(2.2) | 3,247(2.1) | 1,382(2.5) | 697(2.4) | 152(2.6) | 47(1.8) |
| Symptoms Referable to the Respiratory System | 24,675(10.0) | 15,372(9.9) | 6,020(11.1) | 2,476(8.6) | 525(8.9) | 282(10.9) |
| Symptoms Referable to the Digestive System | 37,346(15.1) | 23,030(14.8) | 8,105(14.9) | 4,853(16.8) | 901(15.3) | 457(17.7) |
| Symptoms Referable to the Genitourinary System | 13,552(5.5) | 6,897(4.4) | 3,689(6.8) | 2,351(8.1) | 477(8.1) | 138(5.3) |
| Symptoms Referable to the Skin, Nails, and Hair | 8,040(3.3) | 4,858(3.1) | 1,897(3.5) | 1,001(3.5) | 205(3.5) | 79(3.1) |
| Symptoms Referable to the Musculoskeletal System | 38,773(15.7) | 24,909(16.0) | 8,722(16.1) | 4,070(14.1) | 672(11.4) | 400(15.5) |
| Other | 38,369(15.5) | 26,266(16.9) | 6,615(12.2) | 4,214(14.6) | 881(14.9) | 393(15.2) |

Note: the missing proportion for arrival time, residency type, insurance, and residency type is less than 5%; the missing proportion for temperature, heart rate, and blood pressure is 5–10%; the missing proportion for triage level is 16%, for Seen within last 72 hours is 13%, for pain level is 21%.

Supplement Table 3. Proportion of Emergency Severity Index, Hospital admission, ICU Admission, Medical Resources Utilization, Stratified by Race/Ethnicities, NHAMCS 2005–2016 (Unweighted Sample)

|  | All | White patients | Black patients | Hispanic patients | Asian patients | Other |
| --- | --- | --- | --- | --- | --- | --- |
| **ESI score** |  |  |  |  |  |  |
| 1 – Immediate | 6,499(3.1) | 4,448(3.4) | 1,266(2.8) | 608(2.5) | 131(2.7) | 46(2.2) |
| 2 – Emergent | 26,948(12.9) | 17,480(13.3) | 5,497(12.0) | 2,975(12.5) | 757(15.3) | 239(11.2) |
| 3 – Urgent | 97,634(46.9) | 61,716(46.9) | 20,962(45.8) | 11,446(47.9) | 2,479(50.2) | 1,031(48.3) |
| 4 – Semi-urgent | 59,027(28.4) | 36,851(28.0) | 13,643(29.8) | 6,616(27.7) | 1,258(25.5) | 659(30.9) |
| 5 – Non-urgent | 18,078(8.7) | 10,966(8.3) | 4,409(9.6) | 2,227(9.3) | 316(6.4) | 160(7.5) |
| **Hospital admission** | 42,039(17.0) | 28,681(18.4) | 7,671(14.1) | 4,045(13.9) | 1,236(20.9) | 406(15.6) |
| **ICU** | 4,856(2.0) | 3,334(2.1) | 946(1.7) | 409(1.4) | 121(2.0) | 46(1.8) |
| **Death in ED/hospital** | 1,374(0.6) | 957(0.6) | 249(0.5) | 104(0.4) | 54(0.9) | 10(0.4) |
| **Blood test** | 118,494(47.8) | 75,704(48.6) | 24,561(45.1) | 13,817(47.5) | 3,186(53.8) | 1,226(47.2) |
| **Any image** | 122,247(49.3) | 80,684(51.7) | 23,990(44.1) | 13,342(45.8) | 3,021(51.0) | 1,210(46.6) |
| **Procedure** | 123,247(49.7) | 79,353(50.9) | 24,983(45.9) | 14,252(49.0) | 3,357(56.7) | 1,302(50.2) |
| **Waiting time (minutes, MEANS (95% CI))** | 52.2(51.8-52.5) | 46.1(45.7-46.5) | 65.4(64.5-66.3) | 60.7(59.5-61.9) | 51.2(48.7-53.7) | 49.9(46.7-53.1) |
| **Length of visit (minutes, MEANS (95% CI))** | 232.8(231.7-234.0) | 215.4(214.1-216.7) | 257.0(254.5-259.5) | 279.4(275.2-283.7) | 257.9(250.4-265.4) | 211.4(202.6-220.2) |

Notes: Waiting time: time from arrival to seeing the physician; Length of visit: time from arrival to discharge.
